# The Paneth Cell: The Curator and Defender of the Immature Small Intestine

**DOI:** 10.3389/fimmu.2020.00587

**Published:** 2020-04-03

**Authors:** Shiloh R. Lueschow, Steven J. McElroy

**Affiliations:** ^1^Department of Microbiology and Immunology, University of Iowa, Iowa City, IA, United States; ^2^Stead Family Department of Pediatrics, University of Iowa, Iowa City, IA, United States

**Keywords:** paneth cell, necrotizing enterocolitis, immature intestine, defensins, cathelicidin (LL37), cell death

## Abstract

Paneth cells were first described in the late 19th century by Gustav Schwalbe and Josef Paneth as columnar epithelial cells possessing prominent eosinophilic granules in their cytoplasm. Decades later there is continued interest in Paneth cells as they play an integral role in maintaining intestinal homeostasis and modulating the physiology of the small intestine and its associated microbial flora. Paneth cells are highly specialized secretory epithelial cells located in the small intestinal crypts of Lieberkühn. The dense granules produced by Paneth cells contain an abundance of antimicrobial peptides and immunomodulating proteins that function to regulate the composition of the intestinal flora. This in turn plays a significant role in secondary regulation of the host microvasculature, the normal injury and repair mechanisms of the intestinal epithelial layer, and the levels of intestinal inflammation. These critical functions may have even more importance in the immature intestine of premature infants. While Paneth cells begin to develop in the middle of human gestation, they do not become immune competent or reach their adult density until closer to term gestation. This leaves preterm infants deficient in normal Paneth cell biology during the greatest window of susceptibility to develop intestinal pathology such as necrotizing enterocolitis (NEC). As 10% of infants worldwide are currently born prematurely, there is a significant population of infants contending with an inadequate cohort of Paneth cells. Infants who have developed NEC have decreased Paneth cell numbers compared to age-matched controls, and ablation of murine Paneth cells results in a NEC-like phenotype suggesting again that Paneth cell function is critical to homeostasis to the immature intestine. This review will provide an up to date and comprehensive look at Paneth cell ontogeny, the impact Paneth cells have on the host-microbial axis in the immature intestine, and the repercussions of Paneth cell dysfunction or loss on injury and repair mechanisms in the immature gut.

## Introduction

In the small intestine, intestinal epithelial cells form an important physical and biochemical barrier that prevents the microbial communities contained within the lumen from accessing the rest of the body and causing infection (1). One particular type of intestinal epithelial cell, the Paneth cell, was first discovered by Gustav Schwalbe in the late 19th century based on the eosinophilic granules evident in their cytoplasm. A few years later, Paneth cells were described in depth by their namesake, Joseph Paneth (2, 3). They are now well-recognized as pyramidal shaped, columnar, secretory cells situated at the base of the crypts of Lieberkühn, which are small depressions in the mucosal surface along the small intestine (4). While Paneth cells have occasionally also been found patchily dispersed in the stomach and colon, this is generally associated with mucosal inflammation as opposed to homeostasis (4).

Although Paneth cells were first discovered and described in humans, they are not specific to humans. Paneth cells can be found in many other vertebrates including primates, rodents, horses, sheep, certain fish, and chickens (5, 6). While Paneth cells have been found in this wide variety of other organisms aside from humans, the ontogeny and function are not well-understood for most of them aside from the well-studied and characterized rodents as well as humans. Today, Paneth cells still capture the attention of researchers as they serve an essential role in modulating the microbiome, playing a key part of the innate immune response, and aiding in the proliferation and differentiation of the intestinal epithelium. While Paneth cells have been shown to play important roles in the healthy gut of adults, the development and role of Paneth cells in the immature gut of the preterm infant remains an understudied, but crucial avenue of research that could aid in the understanding of the development of intestinal diseases such as necrotizing enterocolitis (NEC). This review sets out to unveil some of the mystery surrounding Paneth cells in the context of the preterm infant gut and how it relates to NEC.

## The Anatomy of the Paneth Cell

**Figure 1:**
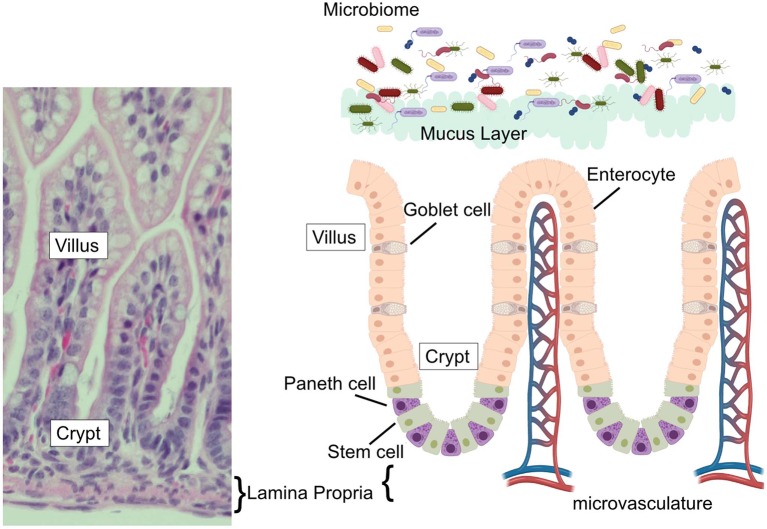
The intestinal epithelium. (Left) H&E stained ileum from P14 C57Bl6 mouse with vilus, crypt, and lamina propria labeled. (Right) Schematic of the intestinal epithelium, associated microbial flora, epithelial cell types (goblet cells, Paneth cells, enterocytes, and stem cells) intestinal microvasculature, and mucus layer. Corresponding labels for vilus, crypt, and lamina propria labeled are placed on the schematic to compare to the H&E stained section.

The human gastrointestinal surface is the largest surface area of the body that is in contact with the external environment (7, 8). This massive surface area is required to allow sufficient nutrient absorption to support growth and health of the host. The small intestine, where Paneth cells reside, has an estimated surface area of 950 cm^2^ at birth, which grows and expands to over 30 m^2^ by adulthood (7, 8). To achieve such a massive surface area, the intestinal surface is clad by fingerlike projections that stick out into the intestinal lumen creating an expansive folding system. This systems' entire surface is covered by a single layer of columnar intestinal epithelial cells (IECs). The intestinal epithelium is the most rapidly-renewing tissue in the adult mammal (9) and undergoes continuous turnover that is generated from Intestinal Stem cells (ISC). The ISC reside at or near the base of the pocket-like intestinal crypts (10, 11) and continuously generate daughter cells that differentiate near the top of the crypts before migrating toward their final destinations. The differentiated cell types are generally grouped by their function as belonging to either the absorptive (enterocytes), or secretory (mucus-secreting goblet, antimicrobial-secreting Paneth, hormone-secreting enteroendocrine cells, and chemosensing/immunomodulatory cytokine-secreting tuft cells) lineage, with clear markers (e.g., *hes1* expression of absorptive and *sox9* for secretory) defining commitment to one or the other arm (12). The typical pattern for these cells is to migrate upwards toward the villus tip in a conveyor-belt-type fashion until they are sloughed off the upper villus into the lumen. However, a unique aspect of Paneth cell biology compared to the other intestinal epithelial cell types is that instead of flowing upward out of the crypt, Paneth cells move downwards further into the crypt as they mature. In addition, while most epithelial cells are rapidly turned over in a few days, Paneth cells can persist for just under 1 month (13). Paneth cell presence is an intestinal priority and their density is rapidly repopulated following their depletion (14). Following their descent into the crypts, Paneth cells are interspersed between the ISCs and can be distinguished by their columnar to pyramidal shape and by the presence of eosinophilic granules within their cytoplasm (Figure 1).

## Paneth Cell Ontogeny and Differentiation

Paneth cells first appear in the small intestine of humans at 13.5 weeks gestational age (15, 16). Paneth cell density in the developing fetal intestine is relatively low, but gradually increases throughout gestation, with significant increases in the third trimester after 29 weeks completed gestation (17, 18). Paneth cell levels do not reach quantities similar to adult levels until term gestation or later (17). Because Paneth cells are located primarily in the distal small intestine, studies using human tissues have been challenging. Thus, much of our understanding of *in vivo* Paneth cell biology has been generated using animal models, predominantly in mice. It is therefore important to note that not all mammals develop Paneth cells prenatally, but instead develop them mid-way through intestinal development after villus development, but before intestinal maturity according to a normal developmental pattern. For example, the commonly used C57Bl/6 mouse strain does not develop Paneth cells until 7–10 days after birth (18, 19).

Paneth cells, like all other intestinal epithelial cell types, are derived from ISCs. In the last decade, it has become clear that ISCs are quite complex. Current models suggest multiple, potentially interconvertible populations of stem cells exist. The first is the crypt-base columnar (CBC) cells (20), slender cells wedged at the very base of the crypt between the Paneth cells. CBC cells carry the specific marker *LGR5* and are actively proliferating (21, 22). The second ISC population express *Bmi1, mTert*, and *Lrig1* markers, and have been hypothesized to be quiescent stem cells until injury occurs, at which time they actively proliferate and produce daughter progeny (23). Interconversion between the two compartments and overlap between the populations has been demonstrated (24). Under normal conditions, the LGR5^+^ ISCs proliferate to generate daughter cells that move out of the crypt. These cells become differentiated as they migrate, and both their differentiation and the maintenance of the stem cells in their proper place is driven through gradients and juxtracrine signaling of Bmp, Wnt, Notch, and growth factor pathways (25, 26). Furthermore, while the exact sources of ligands for these pathways are not fully understood, it is important to note that Paneth cells produce EGF, Notch, and Wnt, which in turn promote stem cell proliferation and maintenance (27). In fact, Paneth cells can support LGR5^+^ cell growth and survival *in vitro*, and have been proposed as a key nurse cell for the actively dividing stem population (27).

**Figure 2:**
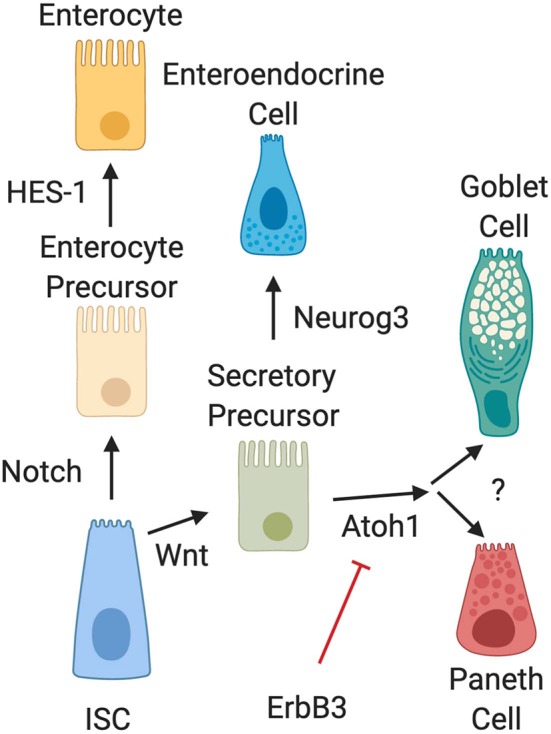
Intestinal epithelial cell differentiation pathways. The intestinal stem cell (ISC) differentiates into absorptive (enterocyte) or secretory precursors through Wnt/Notch signaling. While enterocytes further differentiate through HES-1 signaling, secretory lineages can differentiate into different cell types depending on conditions. Wnt signal pathways drive ISC differentiation into secretory precursor cells. Secretory precursors then develop either into enteroendocrine cells through Neurog3 signaling, or into goblet and Paneth cells following activation of Atoh1. Differentiation signal pathways to separate development of goblet cells and Paneth cells are still unknown. It is also important to note that recent data has shown that activation of ErbB3 acts as a suppressor of Atoh1, while genetic deletion of ErbB3 induces precocious development of Paneth cells.

Several biochemical pathways have been implicated in the development of Paneth cells (Figure 2). Naïve daughter cells are driven to either an absorptive enterocyte phenotype by Notch signaling, or to a secretory phenotype through Wnt signal pathways. The Wnt/β-catenin pathway is an important stimulator of Paneth cell differentiation (28, 29). However, the Wnt signal pathway and its relationship to Paneth cell development is complex and still not completely elucidated. Genetic knockout of LGR-5, a downstream target of Wnt signaling has been shown to produce precocious Paneth cell differentiation in fetal intestine (29, 30). This contradictory data may be due to alterations in negative feedback mediators in the Wnt pathway. Following differentiation into a secretory lineage, activation of the transcription factors Atoh1 (also known as Math1) induces differentiation into a combined goblet/Paneth cell precursor cell lineage (31–35), while genetic ablation of Atoh1 in transgenic mice has been shown to result in loss of Paneth cell lineages (35, 36). Atoh1 has also been shown to be affected by ErbB3, a Receptor Tyrosine Kinase also known as neuregulin (37). Genetic loss of ErbB3 in mice results in unchecked activity of the transcription factor Atoh1 and induces precocious appearance of Paneth cells (37). In addition, activation of ErbB3 can delay normal Paneth cell development. C57Bl/6 mice normally develop Paneth cells by day 10 of life (19). It is however important to note that modifications to Atoh1 signal pathways also affect goblet cell differentiation (36), so understanding of signal pathways that distinguish goblet cell from Paneth cell differentiation downstream of Atoh1 is still incomplete.

## Paneth Cell Role in the Small Intestine

After their migration to the crypt base and subsequent maturation, Paneth cells can be easily distinguished by their prominent acidophilic granules. The granules hold many of the proteins and peptides that Paneth cells secrete to both modulate the microbiome and mediate the inflammatory response. These include: α-defensins (cryptdins in mice), lysozyme, secretory phospholipase A2 (sPLa2), TNF, RegIII, angiogenin-4, MMP-7, CD15, CD95 ligand, xanthine oxidase, IgA, CRIP, metallothionine, adipokines, serum amyloid A, α-1-antitrypsin IL-17A, IL-1β and lipokines (3, 38, 39). These granular components are assembled and packaged by an extensive endoplasmic reticulum (ER) and Golgi apparatus network into dense core granules. (13, 39–43) It is important to note that it is possible that some components of the granules may be produced elsewhere before being collected and added to the granules. IgA is one such component which may be produced by plasma cells in the lamina propria before accumulating and associating in Paneth cell granules (44). Since Paneth cells are not currently able to be cultured without other epithelial and stem cells, most of the data we have on granular contents is from immunohistochemistry techniques. The granules are then released at the apical surface of the cell into the lumen of the intestine where they serve a variety of biological functions, primarily as microbiocidal agents against bacteria, fungi, spirochetes, protozoa, and enveloped viruses (45). Paneth cell granules are secreted both constitutively and in response to pathogenic exposure, with common stimuli including cholinergic stimulation and exposure to bacterial antigens (45–47). This secretion of Paneth cell granular components is under tight regulatory control, as these mediators are vital for maintenance of intestinal homeostasis (38, 48, 49).

Paneth cell health remains a critical priority to the homeostasis of the small intestine. We and others have shown that following dithizone-induced loss, the small intestine replenishes Paneth cell populations within 72 h (14, 50, 51). Since the mammalian intestinal tract represents the largest surface area that communicates with the external environment (7, 52), protection of the host from injury or bacterial invasion from the intestinal flora (53) requires a complex system of defense mechanisms. In the small intestine, a key component of host defense is epithelial derived antimicrobial peptides (AMPs). AMPs are small peptides generally >5 kDa in length, cationic at a neutral pH, and have broad spectrum microbicidal activities at low concentrations (45). These peptides are the main product contained in Paneth cell granules.

**Figure 3:**
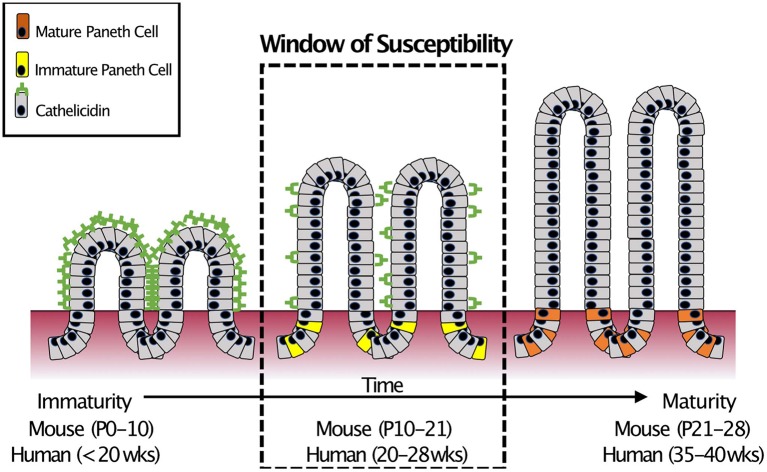
Small intestinal AMP switch during intestinal development. During development, the immature intestine is protected by the AMP CRAMP (LL-37 in humans). However, CRAMP expression decreases around mid-development, at roughly the time that Paneth cells begin to develop. This “switch” occurs during mid-intestinal development which is around post-natal (P) day 10–21 in C57Bl6 mice and in the second trimester (between 20 and 28 weeks of gestation) in humans. In infants born prematurely, this switch is temporally similar to when extremely preterm infants are most susceptible to develop NEC (18).

In humans, there are two major classes of AMPs: cathelicidins and defensins. Cathelicidins are antimicrobial peptides with broad antibacterial (54), anti-fungal (55), and anti-viral activity (56), and are characterized by a highly conserved N-terminal domain. Only after cleavage of the AMP does the protein exert its myriad activities (57). Humans express only one cathelicidin, LL-37 (originally hCAP-18) (58) and it is expressed in various cells of the body including those of the intestinal epithelium (59–62). However, in the small intestine, cathelicidin expression is restricted to the neonatal period (63, 64) before markedly decreasing and disappearing. The timing of this decrease is important as it coincides with the appearance of Paneth cells (19, 65) and the onset of expression of Paneth cell AMPs such as α-Defensins (18). This “switch” from one AMP to another occurs at roughly the mid-point of development of the small intestine (66). It is important to note that mid-development of the small intestine is also around the time when NEC often occurs in infants born extremely prematurely (67) (Figure 3).

The second class of AMP found in the small intestine are defensins. Defensins are abundant in human cells and tissues that are involved in host defense and have two main subtypes: α-defensins, which are found in granule containing cells such as neutrophils and Paneth cells (also known as cryptdins in mice), and β-defensins which are produced by epithelial cells (68–71). Human Paneth cells produce two main α-defensins known as HD-5 and HD-6 (72). In mice, loss of matrilysin (the proteolytic enzyme needed to activate cryptdins) have altered microbiomes and are more susceptible to *Salmonella* infections (73–75). In addition, mice that have been genetically modified to express HD-5 have enhanced resistance to bacterial invasion (74).

AMPs work by inserting themselves into the bacterial membrane and forming pores, which result in the leakage of bacterial cytoplasmic content (76–78). They can also degenerate bacterial cytoplasmic structures and form extracellular net-like structures, which result in bacterial trapping (79). In animal models, AMPs have been shown to preferentially target non-commensal bacteria while sparing commensal normal flora (47, 80). In addition to killing pathogens, AMPs can also influence the immune system through white blood cell chemotaxis (81), activation of dendritic cells (82), and downregulation of immunomodulators such as cortisol (68, 71).

## Paneth Cells and Mechanisms of Cellular Death

Cells of the body undergo death for a multitude of reasons and through various mechanisms. The mechanisms of cell death include apoptosis, necrosis, necroptosis, pyroptosis, and autophagy. While NEC is defined by necrosis of the intestinal tissue, many of these different cellular death pathways have been implicated in the pathogenesis of NEC. Importantly, several of these pathways are also mechanistically tied to Paneth cell biology.

Apoptosis is a normal part of intestinal health that results in disassembly of the cell and, in general, tends to avoid causing inflammation (83). During apoptosis, cells tend to retract pseudopods, condense chromatin (pyknosis), undergo nuclear fragmentation and then experience blebbing of the plasma membrane (84). This contrasts with cellular necrosis where cells experience organelle swelling, extensive vacuole formation, condensation of nuclei, and release of inflammatory cytokines in a passive or accidental manner (83, 84). One type of apoptosis seen in the intestinal epithelial layer is when the epithelial cells move upward from the crypt toward the tip of the villus. Once they reach the tip, cells are sloughed into the intestinal lumen in a process called anoikis, which is a form of apoptosis (84). There is evidence to show that apoptosis is also involved in the cell death experienced by cells in the stem cell region within the small intestinal crypts although the regulation of the process is not well-understood (84). Apoptosis has been shown by multiple investigators to be important in development of NEC (85–89). Additionally, apoptosis is directly relevant to Paneth cell biology and NEC as our lab has shown that NEC-like injury can be induced in mice by delivering diphtheria toxin to *PC-DTR* mice where a human diphtheria toxin receptor has been attached to the cryptdin-2 promoter of Paneth cells (14, 65). When these mice are exposed to diphtheria toxin, all Paneth cells expressing the construct are lysed through apoptotic pathways (90).

Another form of cell death directly related to Paneth cells is autophagy, which is a self-degradative process thought to help remove cells with misfolded or aggregated proteins or other intracellular damage (91). Autophagy is characterized by creation of an intracellular vacuole known as the autophagosome (83). The autophagosome is formed around damaged intracellular organelles or other selected targets. The autophagosome is then fused with a lysosome allowing for degradation of the components within the autophagosome followed by chromatin condensation (83). The morphologic changes that occur tend to be relatively well-regulated similar to the degree of regulation of apoptosis. Also similar to apoptosis, because the degradation of the dying cell takes place within another cell, this process tends to prevent inflammation (83). Autophagy is also an important process for Paneth cells. Because Paneth cells tend to live longer than most other cells of the gut and have many aggregated proteins that could be recycled by other neighboring cells, as damage and stressors to the cells occur, autophagy becomes activated (92). When mutations occur in the autophagy pathway such as in Atg16l1, Paneth cells can become dysfunctional and ultimately trigger intestinal inflammation, which can have implications for gut health such is suggested to be the case with Crohn's disease (92) and NEC (93). Our laboratory has also shown that autophagy may play a role in development of NEC. Lueschow et al. (14) showed that dithizone-induced Paneth cell loss in an experimental murine NEC model resulted in upregulation of autophagy pathways in Paneth cells (14).

Lastly, a more newly described type of cellular death is necroptosis which acts as an intermediate between necrosis and apoptosis. Cells undergoing necroptotic death show features more morphologically similar to necrosis and the immune system creates a highly inflammatory response, but in contrast to necrosis, necroptosis is a well-regulated process, similar to apoptosis (84, 94). Along with this relationship, necroptosis, and apoptosis have a great deal of overlap in their regulation. Apoptosis is promoted by TNFα binding and conversion of the TNFR complex I to the TNFR complex II/alternative TNFR complex (84). Also, the TNFR complex II can regulate as well as induce necroptosis when RIP1 and RIP3 are recruited and deubiquitinated (84). RIP1 and RIP3 are generally under the control of caspase-8, but when an inactivation of the caspase-8 gene occurs, induction of necroptotic cell death ensues although the mechanism by which this occurs is not completely understood (84). Necroptosis is an increasingly important mechanism of cellular death in the intestinal epithelium. Studies have shown that necroptosis of intestinal epithelial cells can result in intestinal inflammation and ultimately produce pathophysiology similar to inflammatory bowel disease (IBD). This was done by creating conditional knockout mice with deletion of FADD or caspase-8, the regulator of necroptosis, in intestinal epithelial cells (54, 95, 96). Interestingly, in addition to induction of necroptosis, this knockout also resulted in spontaneous inflammation and an absence of Paneth cells (84, 95, 96). On further examination, the authors discovered that Paneth cells were uniquely sensitive to necroptosis. This is now thought to be due to the high expression of RIP3, a key modulator of necroptosis, in Paneth cells of humans and mice (84, 95, 96). Necroptosis has also been recently shown to play a role in development of NEC (94). In preterm infants who develop NEC, there is a higher degree of expression in genes related to necroptosis such as *RIPK1, RIPK2*, and *MLKL* compared to preterm infants who do not develop NEC (94). Moreover, increased expression of these three necroptosis related genes was correlated with a greater degree of NEC severity (94). This trend was also observed in murine experimental NEC conditions (94). Overall, these studies highlight the importance of necroptosis as well as Paneth cells in NEC.

## Paneth Cells and Necrotizing Enterocolitis (NEC)

For preterm infants, one of the leading causes of morbidity and mortality, and the most devastating intestinal complication, is development of NEC (97). The incidence of NEC varies widely among developed countries, ranging from 5 to 22% in infants with birth weight <1,000 g (98), and in the US is around 7% (97). Risk factors associated with development of NEC in the preterm infant include degree of prematurity, low birth weight, formula feeding, intestinal ischemia, prolonged antibiotic use, and anemia (99–102). However, the exact etiologic mechanisms and pathophysiology of NEC is still incomplete. In addition, the NEC phenotype may actually be the result of a final common pathway starting from multiple inciting events that results in an imbalance between mucosal injury and epithelial defense and repair, with activation of an unchecked pro-inflammatory cascade (103). As a disease process, NEC is unique in the Neonatal Intensive Care Unit (NICU) population. While the incidence of NEC is directly correlated to the degree of prematurity (the more premature, the more likely to develop NEC), the onset of NEC doesn't happen at birth, but rather weeks after and this delay is longer in the more premature infants. The result is that the incidence of NEC begins to increase at 28 weeks corrected gestational age, peaks at 32 weeks corrected gestational age, and steadily decreases at older corrected gestational ages (67). Theories have been suggested to explain this delay including feeding practices, development of microbial dysbiosis, or the accumulation of mesenteric hypoxic events (99–102). However, there is currently no universally accepted mechanistic explanation. We propose that another plausible reason may be a disruption in the function or quantity of Paneth cells (17, 67, 104).

As discussed above, Paneth cells play a key role in the homeostasis of the small intestinal epithelial lining, and loss or disruption of these cells has been shown to have significant adverse consequences including a reduction in clearance of bacterial pathogens (105, 106), disruption of normal stem cell function (3, 107), and the development of inflammatory bowel disease (108, 109). Paneth cells do not appear in the intestine until approximately halfway through intestinal development and maturation (22–24 weeks of human gestation and P7-10 or mouse age—normal intestinal development in the mouse occurs following birth while in the human it occurs *in utero*) (19, 65, 110). It is also important to note that these early Paneth cells do not possess all the constituents contained in mature granules (65), and it takes weeks in mice and months in humans before the Paneth cell cohort reaches its optimal density and before it becomes fully functional (17). Because of this developmental pattern, premature infants are thus born before they can develop a full complement of functional Paneth cells. As Paneth cells help regulate the intestinal bacterial flora, and NEC requires bacteria to induce intestinal injury, disruption of normal Paneth cell function, especially in the immature intestine could very well be involved in development of the NEC phenotype. Supporting this theory, decreased numbers of lysozyme positive Paneth cells were documented in infants with surgical NEC compared to similar aged surgical controls in two separate studies (111, 112). These data would suggest that Paneth cells are either lost or degranulated during or prior to development of NEC. However, not all studies have shown decreases in Paneth cell function or biology. A study looking at mRNA levels of Human defensin 5 and 6 found that they were increased in infants who developed NEC compared to controls (113). This discrepancy may be explained by timing of surgical resection following the initial Paneth cell disruption. In mouse models, when Paneth cells are disrupted using the heavy metal chelator dithizone, there is an initial decrease in defensin expression followed by a significant increase starting 72 h after treatment (14). In addition, a recent article that examined presence of HD-6 showed a significant decrease following development of NEC (114). Thus, timing of the surgical collection may play a critical role in determining Paneth cell-specific gene expression following NEC.

Studying Paneth cell mechanistic biology in the immature intestine is challenging in humans due to the difficulty of obtaining tissue specimens for preterm infants (115, 116). To help understand the potential role of Paneth cell biology in NEC, several laboratories have instead utilized animal models (100). Interestingly, when Paneth cells are disrupted in neonatal rats followed by enteral exposure to *E. coli*, there is not only an increase in bacterial translocation, but also a development of NEC-like injury to the small intestinal tract (105). In adapting this model to mice, our laboratory and others have shown that selective ablation of Paneth cells followed by enteric gavage of *Klebsiella pneumoniae* in 14-days old mice results in grossly necrotic intestines (89, 117–119), an increase in serum inflammatory markers (119), and alterations in the microbiome (14) that are consistent with human NEC. The use of 2-weeks old mice in this model is potentially advantageous as well as they possess a gene expression profile of epithelial cell genes that matches the expression profile seen in preterm human infants during the window when they are most susceptible to develop NEC (18, 67). Interestingly, disruption of Paneth cell biology via administration of the heavy metal chelator dithizone prior to normal Paneth cell development (5 days old mice) does not result in a NEC-like phenotype (117). One critique of this methodology is that dithizone is not specific to Paneth cells but instead is a general chelator of heavy metals. To help resolve this issue, we developed the *PC-DTR* mouse (14, 119, 120). The *PC-DTR* mouse has a human diphtheria toxin receptor (DTR) inserted into mouse Paneth cells targeting the cryptdin-2 promotor (65). Treatment with diphtheria toxin induces apoptosis of any cells possessing DTR while sparing all other cells. In this model, treatment with diphtheria toxin followed by *Klebsiella pneumoniae* exposure also produces intestinal injury that is equivalent to human NEC (14, 119). These data provide further evidence that it is a disruption of, and not an absence of Paneth cells that contributes to development of NEC-like injury in the immature small intestine.

While these studies show a strong association for Paneth cell disfunction or loss with human NEC as well as a mechanistic relationship in mice, questions about how Paneth cell dysfunction may result in NEC remain (104, 121). It is well-established that prior to the development of NEC there is a dysbiotic change to the microbiome that is marked by a bloom of Proteobacteria, more specifically Enterobacteriaceae species (122–124). This phenomenon has also been replicated in our Paneth cell disruption model of NEC (14). In the normal homeostatic state, the microbiome acts to suppress inflammation through several mechanisms including induction of anti-inflammatory mediators such as IL-10, suppression of pro-inflammatory mediators such as IL-17, and by breaking down and fermenting complex, non-digestible complex polysaccharides into short-chain fatty acids, which possess anti-inflammatory properties (125–127). However, a result of inflammation is increased production of nitric oxide (NO) and superoxide radicals (O${}_{2^{-}}$), which can then react to form nitrates (NO${}_{3^{-}}$). These nitrates can be fermented by facultative anaerobic bacteria such as *Enterobacteriaceae* sp. that belong to the Proteobacteria phyla by utilizing anaerobic respiration with host-derived nitrates as alternative electron acceptors. Since obligate anaerobes cannot use nitrates as a growth substrate, Proteobacteria are able to use this selective pressure to out-compete the obligate anaerobic Firmicutes and Bacteroidetes that rely on fermentation for growth (128). As the proportion of commensal bacteria such as Firmicutes and Bacteroidetes decrease, the production of anti-inflammatory mediators also decreases which further facilitates increased inflammation and dysbiosis. Our laboratory has previously shown that in the immature murine small intestine, exposure to inflammation can significantly decrease the density and function of Paneth cells (129–131).

**Figure 4:**
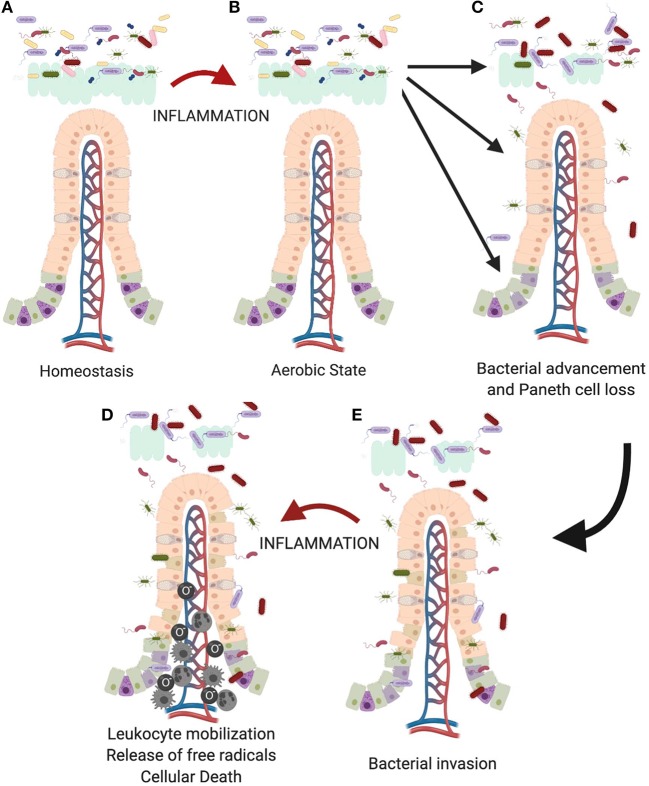
Proposed role of the Paneth cell in development of NEC. As the immature intestine **(A)** is exposed to inflammation **(B)**, oxygen radicals are produced creating a selective advantage for Proteobacteria sp. over obligate anaerobes such as the Firmicutes. This creates a feedback loop for sustaining and increasing the pro-inflammatory state in the immature intestine. Previous work from our lab has shown that intestinal inflammation can reduce intestinal mucus production and cause loss of Paneth cells (112, 129). **(C)** Loss of these important chemical and physical aspects of innate immunity allows bacteria to move from the mucus layer of the intestinal lumen and gain closer proximity to the epithelial surface, **(D)** followed eventually by attachment and invasion of the epithelium. **(E)** Once bacteria invade the intestinal tissue, further inflammation occurs including recruitment of leukocytes including neutrophils, macrophages, and monocytes (133–135) which lead to eventual death of the tissues.

Thus, we think that as the premature infant is exposed to foreign antigens such as formula feedings (132), there is an increase in production of inflammatory cytokines (Figure 4). This creates a more aerobic state leading to a competitive advantage for Proteobacteria, such as Enterobacteriaceae species. As the microbiome becomes more dysbiotic, it suppresses anti-inflammatory mechanisms creating a cycle of increasing intestinal inflammation (136). This increasing inflammation can then impact Paneth cell biology leading to a loss in Paneth cells (14, 129, 137). In an already dysbiotic environment, this combination is exactly the milieu that is modeled in our animal model and predisposes to development of injury. This is further compounded because the Paneth cells present in the immature intestine are not fully mature or functional at a baseline (18). This limited Paneth cell cohort also means that there is a limited capacity for protection via AMPs (40). As Paneth cells are lost, AMP levels will further fall, likely reaching a critical threshold under which bacterial invasion of the epithelial tissue can begin to occur (105). Lastly, it is important to remember that Paneth cell loss may also impact the stem cell niche. A healthy stem cell cohort is critical to induce epithelial restitution following injury as Paneth cells support the stem cell niche through the production of EGF, Notch, and Wnt (27, 88, 104, 138).

In summary, the Paneth cell plays a critical role in many facets of intestinal homeostasis, from regulating the microbiota that closely associate with the epithelium, to maintaining the health of the stem cell niche, to helping to regulate levels of inflammation. Disruption of these secretory cells can have an important effect on the ability of the intestinal epithelium to not only protect itself from foreign invaders, but to promote growth and development of the intestine. These functions are especially critical in the immature intestine of premature infants who have a developing intestinal tract associated with a dysbiotic microbiome. Thus, it is reasonable that Paneth cell disruption has been linked mechanistically to development of NEC-like injury. As mortality rates for NEC remain static, a greater understanding of Paneth cell biology may provide a critical novel pathway to understand the development of NEC.

## Author Contributions

SM and SL contributed equally to the drafting and editing of the work.

### Conflict of Interest

The authors declare that the research was conducted in the absence of any commercial or financial relationships that could be construed as a potential conflict of interest.
